# Morphological and Functional Effects of Ultrasound on Blood–Brain Barrier Transitory Opening: An In Vitro Study on Rat Brain Endothelial Cells

**DOI:** 10.3390/cells12010192

**Published:** 2023-01-03

**Authors:** Jacopo Junio Valerio Branca, Matteo Boninsegna, Gabriele Morucci, Donatello Carrino, Claudio Nicoletti, Ferdinando Paternostro, Massimo Gulisano, Leonardo Bocchi, Alessandra Pacini

**Affiliations:** 1 Department of Experimental and Clinical Medicine, Human Anatomy Section, University of Florence, L.go Brambilla 3, 50134 Florence, Italy; 2 Department of Information Engineering, University of Florence, Via Santa Marta 3, 50139 Florence, Italy; 3 Department of Translational Research and New Technologies in Medicine and Surgery, University of Pisa, 56126 Pisa, Italy

**Keywords:** blood–brain barrier, ultrasound, tight junction, zonula occludens-1

## Abstract

With the recent advances in medicine, human life expectancy is increasing; however, the extra years of life are not necessarily spent in good health or free from disability, resulting in a significantly higher incidence of age-associated pathologies. Among these disorders, neurodegenerative diseases have a significant impact. To this end, the presence of the protective blood–brain barrier (BBB) represents a formidable obstacle to the delivery of therapeutics. Thus, this makes it imperative to define strategies to bypass the BBB in order to successfully target the brain with the appropriate drugs. It has been demonstrated that targeting the BBB by ultrasound (US) can transiently make this anatomical barrier permeable and in so doing, allow the delivery of therapeutics. Thus, our aim was to carry out an in depth in vitro molecular and morphological study on the effects of US treatment on the BBB. The rat brain endothelial (RBE4) cell line was challenged with exposure to 12 MHz diagnostic US treatment for 10, 20, and 30 min. Cell viability assays, Western blotting analysis on the endoplasmic reticulum (ER), and oxidative stress marker evaluation were then performed, along with cytological and immunofluorescence staining, in order to evaluate the effects of US on the intercellular spaces and tight junction distribution of the brain endothelial cells. We observed that the US treatment exerted no toxic effects on either RBE4 cell viability or the upregulation/dislocation of the ER and oxidative stress marker (GRP78 and cytochrome C, respectively). Further, we observed that the application of US induced an increase in the intercellular spaces, as shown by Papanicolaou staining, mainly due to the altered distribution of the tight junction protein *zonula occludens*-1 (ZO-1). This latter US-dependent effect was transient and disappeared 20 min after the removal of the stimulus. In conclusion, our results show that US induces a transient alteration of the BBB, without altering the intracellular signaling pathways such as the ER and oxidative stress that could potentially be toxic for endothelial cells. These results suggested that US treatment could represent a potential strategy for improving drug delivery to the brain.

## 1. Introduction

Neurodegeneration is a term encompassing a range of neurological disorders affecting the central nervous system (CNS), such as dementia, Alzheimer’s disease, Huntington’s disease, and Parkinson’s disease [1]. The mechanism(s) underlying neurodegeneration remain to be determined; however, it is generally accepted that neurological disorders can arise from a variety of factors, including physiological aging, chronic inflammation, and toxicant exposure [2,3,4,5].

From a therapeutic point of view, the treatment of these dramatic disorders of the CNS is hampered by the presence of the blood brain barrier (BBB), a highly efficient anatomical barrier that prevents the delivery of the appropriate drugs to the brain [6].

Indeed, the BBB is a defense system that supplies the brain parenchyma with oxygen and nutrients while at the same time, blocking the entry of toxic substances [7]. The BBB microvessels consist of endothelial cells that are strictly sealed by each other, thanks to the presence of tight junction (TJ) proteins [8], which only allow highly selected substances to have access to brain parenchyma [9]. Thus, invasive and non-invasive methods have been evaluated in order to identify effective drug delivery systems to target specific areas of the brain [9,10]. To this end, the use of ultrasound (US) has been shown to be a promising approach [11].

Ultrasound is a non-invasive imaging technique mainly used in medicine to perform both diagnostic and therapeutic procedures. Indeed, it has been demonstrated that US, thanks to its mechanical vibrations, could be used to positively influence neurons and microglia physiology [11,12,13,14], without damaging or increasing the temperature of the involved tissues [15,16,17]. Hameroff et al. demonstrated that transcranial US has beneficial effects on the psychological aspects of chronic pain patients [11]. Moreover, in vitro experiments showed US-dependent neurites sprouting and increase in length and number of cytoplasmic elongations [18,19,20].

It is therefore not surprising that US has been widely used to noninvasively induce localized BBB openings in specific areas of the brain as a drug delivery system, bypassing the BBB [21,22,23,24].

Despite the multiple in vivo and in vitro demonstrations of US effects, very little is known about the US-induced molecular and morphological mechanism triggered in endothelial cells of the BBB; this prompted us to investigate the effects of US on a rat brain endothelial cell line (RBE4), a well-established BBB in vitro model [25,26,27].

## 2. Materials and Methods

### 2.1. Cell Culture and Treatments

The BBB monolayer model, the rat brain endothelial (RBE4) cell line (kindly provided by Dr. Vincenzo Giuseppe Nicoletti—Dept. of Biomedical Sciences, University of Catania, Catania, Italy) was used. The cells were cultured in alpha-minimal essential medium (alpha-MEM)/Ham’s F10, supplemented with 10% fetal bovine serum (FBS), 1 ng/mL basic fibroblast growth factor (bFGF), 1% penicillin/streptomycin (Thermo Fisher Scientific, Milan, Italy) at 37 °C, 5% CO_2_, in a humidified atmosphere. The US stimulation was executed using the MyLab ultrasound device (ESAOTE, Florence, Italy) plugged to the linear probe LA523 at 12 MHz of frequency.

In order to reduce the US signal attenuation caused by air, an ultrasound transmission gel (Aquasonic 100, Parker Laboratories, Fairfield, NJ, USA) was used. Preliminary tests were carried out to assess the ability of the US beam to cross a plastic medium and the glass coverslip, as previously reported [18,19]. The frequency of 12 MHz allows the penetration of the US in the medium up to about 2 cm. This value was greater than the cumulative thickness of polystyrene dish and cellular layer. The RBE4 were either cultured in complete growth medium on a square (22 mm × 22 mm) coverslip at 8 × 10^4^ cell density, or on Petri dishes (Ø = 100 mm) at 3.5 × 10^6^ cells. The different supports were used accordingly to the different evaluation performed, as reported in the Materials and Methods (Section 2.3, Section 2.4 and Section 2.5). When the experiments were performed on Petri dishes, one randomly chosen half of the Petri dish underwent the US treatment, whereas the other half was used as an internal control. When cells were seeded on square cover glasses, the US linear probe was aligned in the central part of the coverslip, while the lateral regions were used as the untreated, internal control group. The length of the US stimulation was set at 10, 20 and 30 min at the same frequency, according to the method used in a previously published report [28]. The exposure to ultrasound was chosen according to the results of previous experiments (not shown) at earlier exposure times (1, 3, and 6 min), but no significant changes were observed. The experiments were performed in starvation medium.

### 2.2. MTT Assay

RBE4 cells (2 × 10^4^ cells/well) were plated on 96-well plates and incubated in complete medium for 24 h to reach confluence. After 24 h starvation, cells were stimulated by ultrasonic radiation, as previously described [18,19]. A potential US cell cytotoxicity was assessed by spectrophotometrically measuring mitochondrial reductase activity using the MTT [3-(4,5-dimethylthiazol-2-yl)-2,5-diphenyltetrazolium bromide] assay kit, according to the manufacturer’s instructions (Sigma Aldrich, cat n. M2003, Milan, Italy).

Absorbance values were measured by a spectrophotometer (MultiskanFC™ microplate photometer, Thermo Fisher Scientific, Milan, Italy) set to a 570 nm wavelength.

All experiments were repeated three times in quintuplicate.

### 2.3. Western Blotting Analysis

After US stimulation, Western blotting analysis was performed. Equal amounts of proteins (30 µg) were separated on 12% SDS-PAGE gels and transferred to nitrocellulose membranes (Porablot NPC, MACHEREY-NAGEL, Milan, Italy). After blocking in 5% BSA (bovine serum albumin) for 1 h at room temperature (RT), the membranes were incubated overnight at 4 °C with primary antibodies against GRP78 (1:500; Thermo Fisher Scientific, cat. n. PA1014A, Milan, Italy) and β-actin (1:10000; Santa Cruz Biotechnology, cat. n. sc-47778, Milan, Italy). Detection was performed with the appropriate HRP-conjugated secondary antibodies (1:5000; Santa Cruz Biotechnology, cat. n. sc-2004 and sc-2005, Milan, Italy) and enhanced by chemiluminescent substrates (ECL Plus Western Blotting Detection Reagent, GE Healthcare, Milan, Italy). All assays were performed in triplicate.

The band density was determined using ImageJ software (ImageJ, National Institute of Health, Bethesda, MD, USA, http://imagej.nih.gov/ij, 1.53f) and normalized by β-actin as an internal loading control.

### 2.4. Papanicolaou Cytological Staining and Intercellular Spaces Evaluation

RBE4 cells were cultured in complete growth medium on square (22 mm × 22 mm) coverslips at 8 × 10^4^ cell density. The steps related to starvation and treatment are the same as those described above.

After US stimulation, the specimens were fixed with 0.5% paraformaldehyde for 10 min at RT and subsequently washed twice in PBS.

The quantitative analysis of intercellular spaces was performed on RBE4 cells using a routinely used Papanicolaou staining, without the use of the Orange G6 step, in order to obtain the greatest contrast between the stained cells and the background.

**Figure 1:**
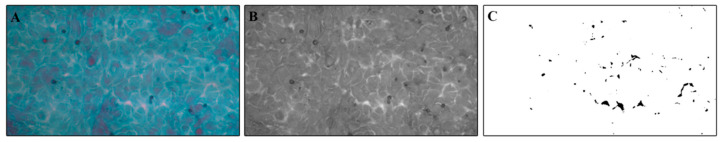
ImageJ threshold tool for intercellular spaces analysis on Papanicolaou staining. The original images (panel **A**) were first converted to black and white 8-bit images (panel **B**), and subsequently, the threshold tool was used (panel **C**).

Finally, the specimens were mounted on microscope glass slides with Canada balsam. All reagents used were purchased from Sigma Diagnostics (St. Louis, MO, USA). Each sample was examined by an optical microscope (Zeiss Axioskop 20; Carl Zeiss S.p.A., Milano, Italy) at different magnifications, and images were acquired with a digital photo camera (Truechrome HD, TiEsseLab S.r.l., Milano, Italy). The intercellular spaces were measured by ImageJ software (ImageJ, National Institute of Health, Bethesda MD, USA, http://imagej.nih.gov/ij, 1.53f), converting the image in black and white, and using the threshold mode, as reported in Figure 1.

Five microscopic fields were randomly selected for each experiment at different time points; each time point was performed in triplicate.

### 2.5. Immunofluorescent Labeling

RBE4 cells were seeded on sterilized coverslips, as described in the Papanicolaou staining section. After US stimulation, the cells were fixed with cold methanol for 20 min for *zonula occludens*-1 (ZO-1), or with 4% paraformaldehyde for 10 min at RT for cytochrome C and F-actin. After permeabilization with 0.1% Triton X-100 for 10 min and blocking nonspecific binding in 1% BSA for 30 min, the cells were incubated with rabbit anti-ZO-1 (1:50; Thermo Fisher Scientific, cat. n. 402200, Milan, Italy) or anti-cytochrome C (1:200; Santa Cruz Biotechnology, cat. n. sc-13156, Santa Cruz, CA, USA) primary antibodies overnight at 4 °C. F-actin was stained using Alexa-488 conjugated phalloidin (1:200; Thermo Fisher Scientific, cat. n. A12379, Milan, Italy). Alexa-568 or -488 conjugated donkey anti-rabbit or donkey anti-mouse IgG secondary antibodies (1:200; Invitrogen, cat. n. A10042 and A21202, Milan, Italy) were used to reveal the immunopositive cells. Cellular nuclei were stained with DAPI (4′,6-diamidino-2-phenylindole; 1:2000 dilution; Invitrogen, Milan, Italy). Five microscopic fields were chosen for each experimental point at 400× total magnification using a motorized Leica DM6000B microscope equipped with a DFC350FX camera. Each experimental point was performed in triplicate.

F-actin immunostaining was conducted, measuring the peripheral edge and the internal area using ImageJ software (ImageJ, National Institute of Health, Bethesda, Montgomery, MD, USA, http://imagej.nih.gov/ij, 1.53f), and both surfaces were normalized by the whole cell area.

### 2.6. Statistical Analysis

The one-way analysis of variance (ANOVA) for assessing significant differences was performed in all the evaluations, since the performed experiments involved comparison between two homogeneous groups (treated-untreated). Differences were considered statistically significant when *p* < 0.05.

## 3. Results

### 3.1. Cell Viability Assay

**Figure 2:**
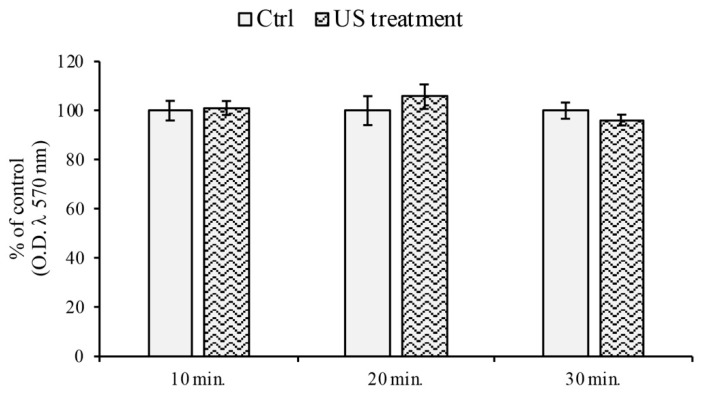
Cell viability (MTT) assay on RBE4 cells during US stimulation. The RBE4 cell line was stimulated with US for 10, 20, and 30 min. As reported, the cell viability did not significantly change during treatment in comparison to those in the control (untreated cells), arbitrarily taken as 100%. Values are expressed as the mean ± S.E.M. of three independent experiments, performed in quintuplicate.

In order to verify whether US stimulation altered the brain endothelial cells viability and metabolic activity, MTT assays on the RBE4 cells, with and without US treatment, were performed at different exposure times. As reported in Figure 2, the US application did not alter the cell viability at any time following US treatment.

### 3.2. Endoplasmic Reticulum and Oxidative Stress Evaluation

**Figure 3:**
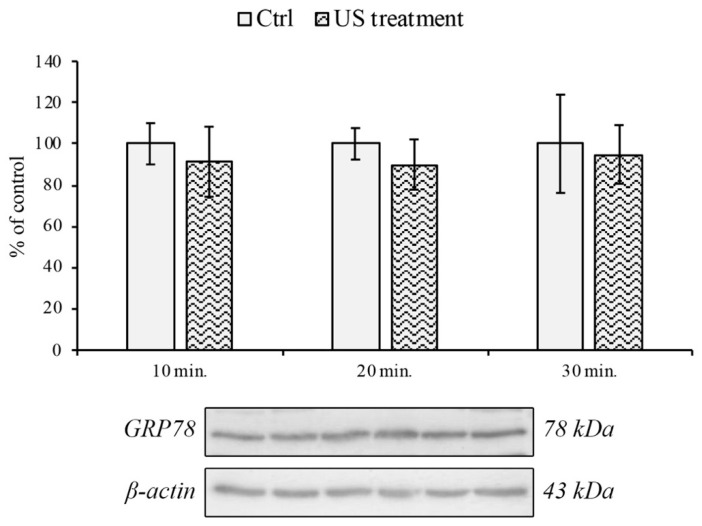
GRP78 protein expression on RBE4 cells during US treatment. Western blotting analysis and quantification of the ER stress marker GRP78 on the RBE4 cell line during US stimulation at 10, 20 and 30 min. time-points. Values are reported as the percentage of the control (untreated cells), arbitrarily taken as 100%. The results are expressed as the mean ± S.E.M. of three independent experiments, performed in triplicate.

The safety of the US treatment on RBE4 was further assessed by monitoring the expression of the endoplasmic reticulum (ER) stress marker GRP78 [29] by Western blotting analysis. In Figure 3, we show that the exposure to US did not alter the expression of GRP78 protein.

**Figure 4:**
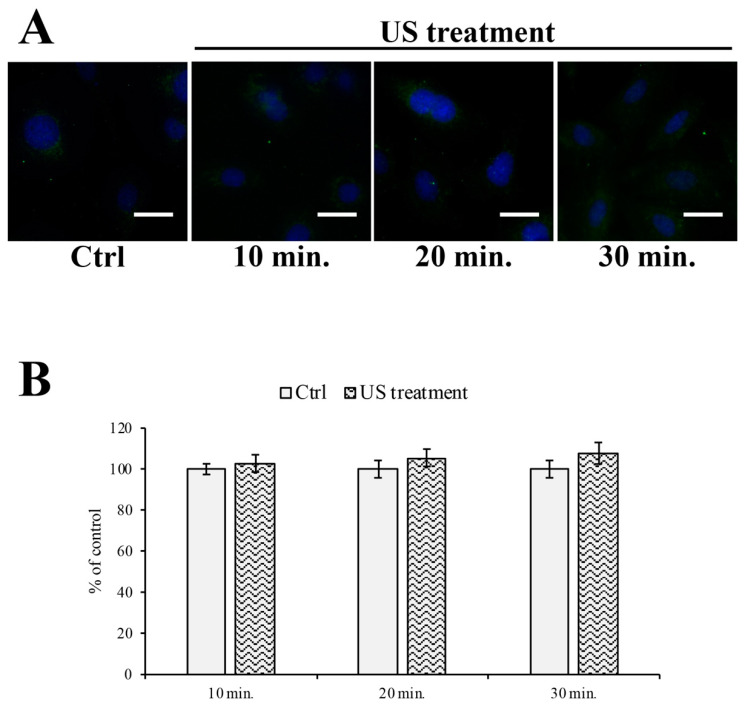
Mitochondrial cytochrome C localization during US treatment. (**A**) Immunofluorescence staining analysis of cytochrome C (green), counterstained with DAPI (blue) during US stimulation for 10, 20, and 30 min, showing a punctate immunostaining pattern of cytochrome C. (**B**) The semi-quantitative analysis of fluorescence levels did not show any differences between the treated samples and the control (untreated cells). Values are reported as percentages of the control (untreated cells), arbitrarily taken as 100%. The results are expressed as the mean ± S.E.M. of three independent experiments, performed in triplicate. Five microscopic fields per treatment have been recorded. Total magnification: 400×; scale bar: 25 μm.

Moreover, we evaluated the expression level of cytochrome C as an oxidative stress marker [30]. Our results demonstrated that the US treatment did not induce the cytochrome C spillage from the mitochondrial subcellular compartment into the cytoplasmic region. However, although the data were not statistically significant, it would appear that the cytoplasmic level of cytochrome C tends to raise with an increasing time of exposure to US (Figure 4).

### 3.3. Evaluation of Intercellular Spaces

**Figure 5:**
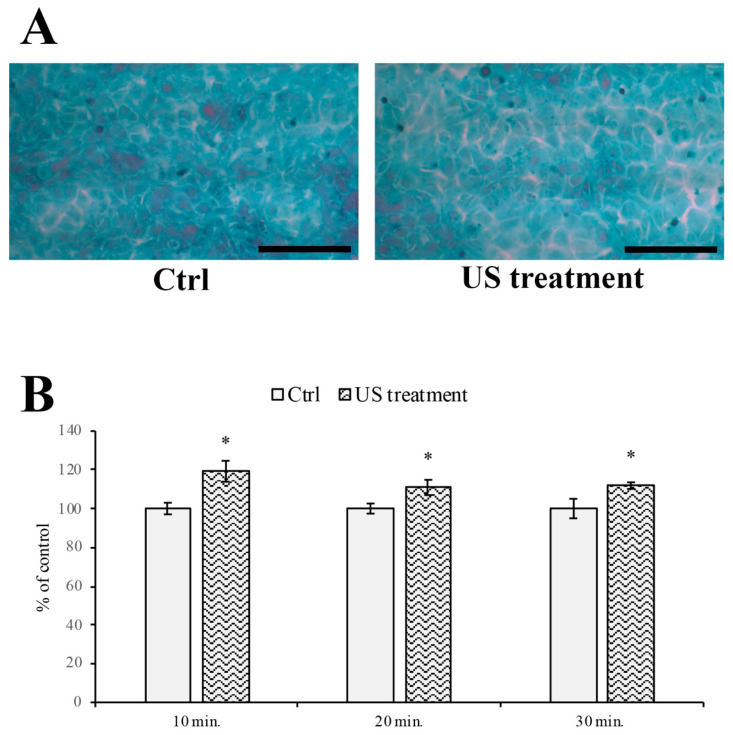
Intercellular spaces analysis on RBE4 monolayer during US treatment. (**A**) Representative illustrations showing the RBE4 monolayer treated with Papanicolaou staining, in both natural condition and after US stimulation. (**B**) Histograms showing the width of the intercellular gaps after US treatment for 10, 20, and 30 min. The results are expressed as the mean ± S.E.M. of three independent experiments, performed in triplicate. Five microscopic fields per experimental point were taken and analyzed. The control (untreated cells) were arbitrarily taken as 100%. Total magnification 200×; scale bar: 100 µm; * *p* ≤ 0.05 vs. control (untreated cells).

The BBB permeability was evaluated by Papanicolaou staining (Figure 5A), offering the greatest contrast between the cells (light blue) and the background (white), allowing for the evaluation of the width of the intercellular spaces. As reported in Figure 5B, the US treatment increases the intercellular spaces between the RBE4 monolayer cells, starting at the 10 min stimulation time point.

### 3.4. F-actin and Tight Junction ZO-1 Distribution Analysis

**Figure 6:**
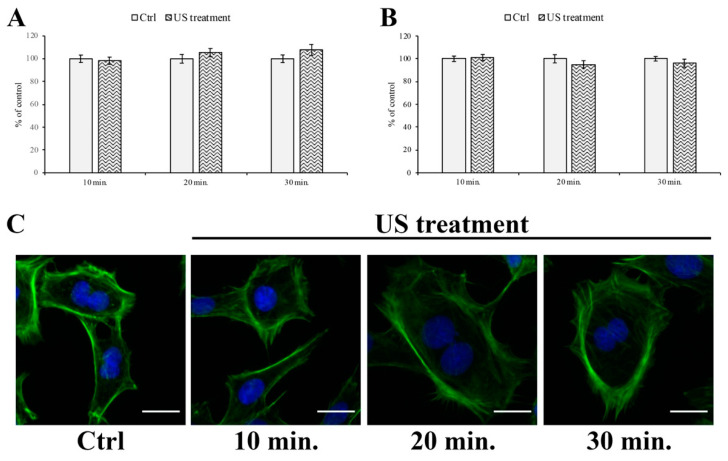
F-actin microfilament distribution and localization during US treatment on RBE4 cells. The microfilament distribution was not significantly altered during US treatment, even if a slight increase in peripheral F-actin surface appeared between 20 and 30 min of stimulus (panel **A**), along with a small opposite decrease in the background actin filament (panel **B**). Values are reported as percentages of the control (untreated cells), arbitrarily taken as 100%. The results are expressed as the mean ± S.E.M. of three independent experiments, performed in triplicate. Representative immunocytofluorescent images at different times of US stimulation were reported (panel **C**). Five microscopic fields per treatment have been recorded. Total magnification 400×; scale bar: 25 µm.

The US-dependent alteration of the F-actin distribution was evaluated after 10, 20, and 30 min of US treatment. As reported in Figure 6, the peripheral F-actin distribution seems to slightly increase the area after 20 and 30 min of US stimulation (Figure 6A). On the other hand, the internal area seems to decrease after 20 and 30 min (Figure 6B).

**Figure 7:**
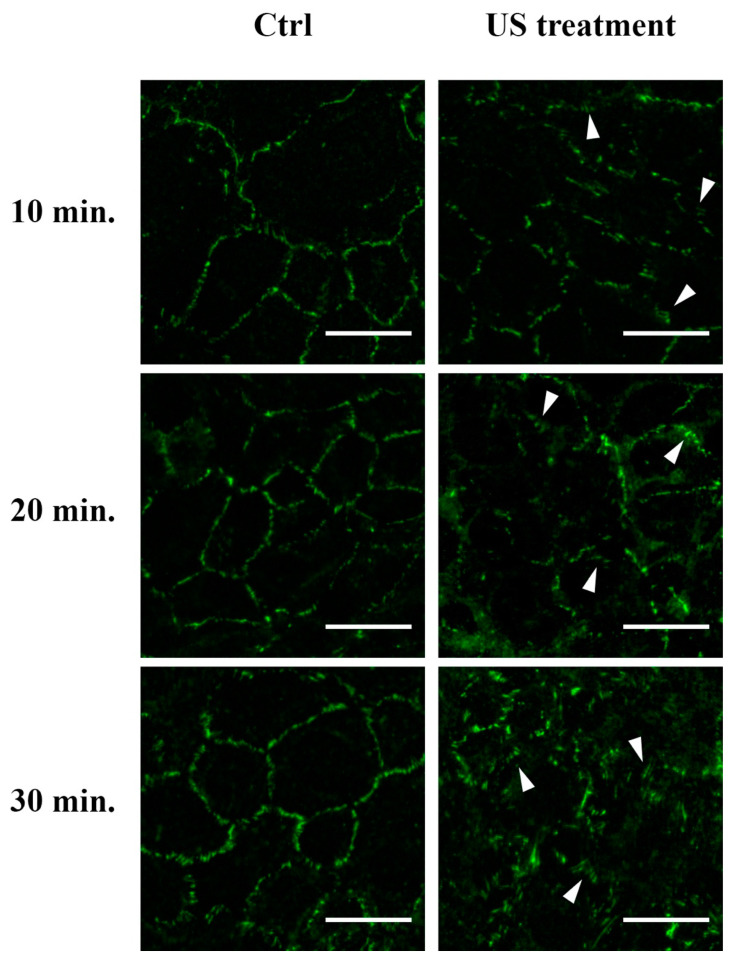
Tight junction ZO-1 localization and distribution on RBE4 cell during US stimulation. The RBE4 cell line was stimulated with US for 10, 20, and 30 min. A clear ZO-1 altered distribution was seen starting at 10 min of US stimulation, highlighting an increased “zip-like” morphology (arrowhead). Five microscopic fields per treatment have been recorded. Total magnification 400×; scale bar: 25 μm.

Finally, we examined the morphological distribution of the tight junction ZO-1 in the RBE4 monolayer cells during US stimulation and in the unstimulated controls. As shown in Figure 7, the US application induced an altered localization of ZO-1 that appeared as a “zip-like” structure, in comparison to untreated controls.

**Figure 8:**
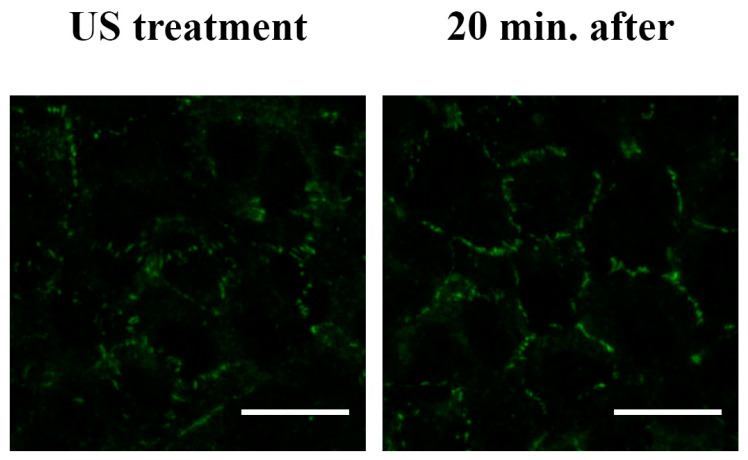
Restoration of the ZO-1 subcellular localization in the absence of US stimulation. The RBE4 cells were treated for 20 min with US, and the distribution of ZO-1 was evaluated 20 min after the removal of the treatment. The ZO-1 distribution clearly shows an unaltered localization, with no significant differences in comparison to the control (untreated cells) (Figure 7). Five microscopic fields per treatment have been recorded. Total magnification 400×; scale bar: 25 μm.

However, the analysis of the dislocation of this TJ, carried out at different times after the removal of the US stimulus, showed a restoration of its morphology comparable to that of the control (untreated cells) (Figure 8).

**Figure 9:**
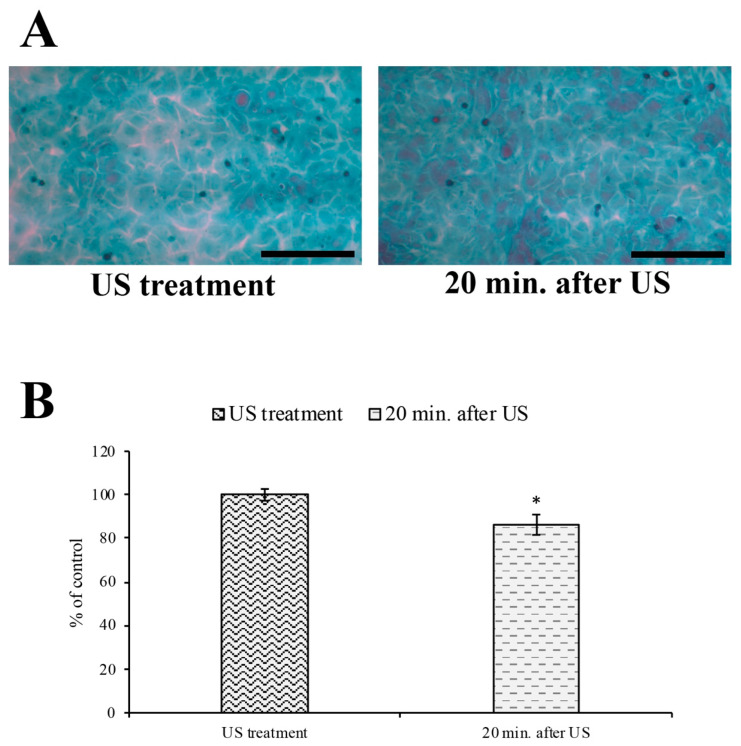
Intercellular spaces analysis on RBE4 monolayer after US removal. (**A**) Representative illustrations showing the RBE4 monolayer treated with Papanicolaou staining after 20 min of US treatment and an additional 20 min after its removal. (**B**) The histogram shows the intercellular spaces (lacuna, gaps) between cells. The results are expressed as the mean ± S.E.M. of three independent experiments, performed in triplicate. Five microscopic fields per experimental point were taken and analyzed. The US treatment cells were arbitrarily taken as 100%. Total magnification 200×; scale bar: 100 µm; * *p* ≤ 0.05 *vs*. US treatment.

This result was also confirmed by the cytological staining analysis (Figure 9), where the intercellular gaps induced by US stimulation were significantly reduced 20 min after US removal.

## 4. Discussion

In recent decades, the increased life span has led to a significant increase in the incidence of significant neurodegenerative disorders such as Alzheimer’s and Parkinson’s disease [31]. Over the years, many strategies and therapeutic approaches have been attempted in order to ameliorate or counteract these adverse events. One of the main obstacles to the delivery of effective doses of specific therapeutics to the brain is the presence of the BBB [32]. On one hand, this anatomical barrier protects the brain parenchyma from interacting with potentially harmful molecules, while on the other hand, it also restricts the entry of most pharmaceuticals into the brain. To overcome this problem, many strategies have been devised, ranging from nanocarriers, as liposomal-based approaches, to US application [33,34,35].

US is well known in the medical field, especially for its diagnostic use [36] and, in recent years, for its role in affecting neuronal functions [11,37,38]. Additionally, many studies, both in vitro and in vivo, have demonstrated the efficacy of US in increasing BBB permeability in order to facilitate drug delivery to the brain. Although considered safe, with different biological effects [39,40], it has been shown that US treatment can affect cell viability via alteration of the cellular membrane [41]. However, this latter effect could be minimized by the US conditions used and the use of specific precautions, such as frequency control and the chelation of intracellular Ca^2+^ [42]. Since the precise mechanisms leading to US-mediated opening of the BBB remain poorly understood, we sought to investigate the US effects using a rat brain endothelial (RBE4) cell line as a BBB in vitro model [43].

It was demonstrated that cell viability was not only dependent on the device specifications (voltage, temperature, exposure time), but also on cell type. Indeed, small changes in temperature significantly affected cell stress and cytotoxicity [44]. In order to assess whether our US experimental conditions altered cellular viability *per se*, RBE4 underwent an MTT assay following exposure to US. We observed that our device specifications had no effect on the viability of RBE4 cells at any time point taken into consideration, confirming previous data obtained using the same US settings on different cell types [24,25]. As previously described, endothelial cells experience shear stress associated with the blood flow [45]. Such a phenomenon is considered to be the main cause of the stress-related expression of genes involved in cell signaling, function, and structure [46] and the formation of reactive oxygen species (ROS) [47]. To this end, it is worth highlighting that US treatment itself has been reported as triggering the shear stress response resulting in ROS formation [48]. This prompted us to monitor the US-mediated shear stress response by measuring the mitochondria release of cytochrome C [49], a marker of oxidative stress. Our data suggested that US did not induce the shear stress response. This is in contrast with the observations by others, although this discrepancy could be explained due to different US properties. Indeed, in our experiments, we used 12 MHz frequencies instead of the commonly used 1–5 MHz [48,50,51]. Moreover, US treatment has been reported to trigger the ER-stress response, leading to the production of GRP78 [49]. In this case, we failed to observe an increase in GRP78 protein expression, thus confirming that under our experimental conditions, US treatment did not induce detectable ER stress.

US treatment induced an increase in the intercellular spaces. Indeed, Papanicolaou staining showed that intercellular gaps between cultured RBE4 cells significantly increased at all time points tested following cell exposure to US treatment. This is in agreement with a previous observation describing US-dependent cytoskeleton alterations [52]. In particular, the appearance of F-actin stress fibers in the center of sonoporated cells has been described [52]. However, in contrast to these data, the immunofluorescent analysis of F-actin in US-treated RBE4 cells failed to confirm these observations. Indeed, a trend towards an increase in the presence of F-actin stress fibers was observed at 20 min post US exposure. However, even if this increase was not statistically significant, it is still present at 30 min. The transient nature of this event might explain these apparently conflicting results. However, we believe this transient event might also impact the US-mediated modification of the distribution of the TJ protein ZO-1. Indeed, the US-related stretching of the F-actin microfilament appeared to be dragging the ZO-1 bound to the cytoskeleton microfilaments [53,54]. The altered distribution of the ZO-1 is likely to result in an increased BBB permeability.

Finally, it is important to note that 20 min after the removal of the US stimulus, the endothelial barrier regained its original morphology, as indicated by the restoration of the ZO-1 distribution and reduction in the intercellular gaps.

## 5. Conclusions

These data demonstrate that US treatment induced a transient and reversible increase in intercellular spaces, without altering cell viability and/or triggering potentially harmful signaling pathways. Although the effects of a US-dependent altered permeability of the BBB are not yet well known and further investigation on the mechanisms underlying BBB opening has yet to be examined, we interpret this data as showing that US could represent a potential tool to deliver safe and efficient therapeutics across the BBB in clinical applications.

## Data Availability

Not applicable.
